# Covariation and repeatability of male mating effort and mating preferences in a promiscuous fish

**DOI:** 10.1002/ece3.607

**Published:** 2013-05-31

**Authors:** Jean-Guy J Godin, Heather L Auld

**Affiliations:** Department of Biology, Carleton UniversityOttawa, ON K1S 5B6, Canada

**Keywords:** Courtship, guppy, male mate choice, phenotypic variation, *Poecilia reticulata*, sexual selection

## Abstract

Although mate choice by males does occur in nature, our understanding of its importance in driving evolutionary change remains limited compared with that for female mate choice. Recent theoretical models have shown that the evolution of male mate choice is more likely when individual variation in male mating effort and mating preferences exist and positively covary within populations. However, relatively little is known about the nature of such variation and its maintenance within natural populations. Here, using the Trinidadian guppy (*Poecilia reticulata*) as a model study system, we report that mating effort and mating preferences in males, based on female body length (a strong correlate of fecundity), positively covary and are significantly variable among subjects. Individual males are thus consistent, but not unanimous, in their mate choice. Both individual mating effort (including courtship effort) and mating preference were significantly repeatable. These novel findings support the assumptions and predictions of recent evolutionary models of male mate choice, and are consistent with the presence of additive genetic variation for male mate choice based on female size in our study population and thus with the opportunity for selection and further evolution of large female body size through male mate choice.

## Introduction

Because of sexual differences in parental investment and potential rate of reproduction, males have traditionally been regarded as being indiscriminate and competing for choosy females (Clutton-Brock and Vincent 1991; Andersson 1994; Clutton-Brock 2007; Edward and Chapman 2011). However, accumulating evidence suggests that mate choice by males is relatively common in nature (reviewed in Amundsen 2000; Bonduriansky 2001; Clutton-Brock 2007; Edward and Chapman 2011). Despite recent advances (reviewed in Amundsen 2000; Bonduriansky 2001; Clutton-Brock 2007; Edward and Chapman 2011), our understanding of the evolution and maintenance of male mate choice and its importance in driving evolutionary change remains limited compared with that for female mate choice. Theoretically, male mate choice can evolve under a wider range of conditions than predicted by parental investment or reproductive potential alone (Bonduriansky 2001; Wedell et al. 2002; Servedio and Lande 2006; Clutton-Brock 2007; Edward and Chapman 2011; South et al. 2012). Conditions favoring its evolution include sperm competition among males, female attractiveness for males exhibiting high courtship effort, and variation in female quality, male mating effort and costs of mate choice. For male mate choice to evolve, choosy males must accrue benefits that offset the costs of choice (Parker 1983; Edward and Chapman 2011).

Mate choice by an individual results from the interaction between its mating effort (investments in the sexual pursuit and attraction of prospective mates and in mating) and mating preference (differential ranking or choosing of prospective mates; Jennions and Petrie 1997; Edward and Chapman 2011). More specifically, increased investment in mating effort by a male increases his ability to attract multiple females, but consequently reduces his capacity to mate with those females, thus selecting for male mate choice (Edward and Chapman 2011). Males may therefore exert premating choice by allocating more mating effort (including courtship) toward, and by accepting or rejecting, certain females over others (Edward and Chapman 2011).

Because of fluctuating selection and varying benefits and costs of mating behavior in different environments, phenotypic plasticity in mating effort and mating preference are expected to be common and potentially beneficial for males (Qvarnström 2001; Bretman et al. 2011; Edward and Chapman 2011). If any male experiences greater competition for mates as a consequence of his preferring the same females as other males in the population, then males with either weak or alternative preferences will be at a sexually competitive advantage (Servedio and Lande 2006). Such male–male competition does not favor the evolution of a single shared male preference, but rather selects for individual variation in mating preferences (Servedio and Lande 2006; Edward and Chapman 2011). Evolution of mate choice is therefore more likely when individual variation in mating effort and mating preferences exist within populations (Servedio and Lande 2006; Rowell and Servedio 2009; Edward and Chapman 2011; South et al. 2012). Such variation can have major consequences for sexual selection and is of fundamental importance to the evolution of mate choice (Jennions and Petrie 1997; Widemo and Sæther 1999; Servedio and Lande 2006; Rowell and Servedio 2009; Edward and Chapman 2011; South et al. 2012). Therefore, understanding variation in mating effort and preference is critical for understanding both sexual selection and how diversity arises in nature (Jennions and Petrie 1997; Widemo and Sæther 1999). Within-population variation in female mating preferences has been documented to some extent, and its implications for the evolution of choice in females and elaborate sexual traits in males are widely recognized (e.g., Andersson 1994; Bakker and Pomiankowski 1995; Jennions and Petrie 1997; Widemo and Sæther 1999; Clutton-Brock 2007). This is not yet the case for male mate choice (but see Bel-Venner et al. 2008, for example).

A commonly used measure of the variance structure of any phenotypic trait in a population is its repeatability, which represents the proportion of the total variation in the trait that can be attributable to differences between individuals (Boake 1989; Widemo and Sæther 1999). Repeatability is a measure of the within-individual consistency of the trait over time and is obtained from repeated measures on the same individuals. In quantitative genetics, repeatability of a trait is often used as an upper-bound estimate of its broad-sense heritability (i.e., fraction of total phenotypic variance that is genetic in basis) and thus its responsiveness to selection (Lynch and Walsh 1998). Notwithstanding its limitations (Jennions and Petrie 1997; Widemo and Sæther 1999; Dohm 2002), measuring the repeatability of mating preferences is a first step toward understanding how much preferences vary within a population (Widemo and Sæther 1999). Relatively little information is available on the repeatability of mating preferences expressed by males (Bell et al. 2009) and almost nothing is known about their heritability (Bakker and Pomiankowski 1995; Jennions and Petrie 1997; Chenoweth and Blows 2006).

To improve our relatively limited understanding of variation in male mate choice within natural populations, we characterize here both within- and between-subject variation in mating effort and mating preference directed toward females based on body size, and provide repeatability estimates for them, in wild-caught male Trinidadian guppies (*Poecilia reticulata*) under standardized laboratory conditions. Because a positive relationship between mating effort and mating preference for a particular female trait among males (Servedio and Lande 2006; Rowell and Servedio 2009; Edward and Chapman 2011), and female preference for males exhibiting high courtship effort (South et al. 2012), can in theory favor the evolution of male mate choice, we additionally tested for this relationship in male guppies. We used female body length as the target trait for male choice because it is a reliable proxy of female quality (highly correlated with fertility or fecundity) in the guppy (Reznick and Endler 1982; Kelly et al. 1999; Herdman et al. 2004; Ojanguren and Magurran 2004) and in many other species (Edward and Chapman 2011). Male guppies (Dosen and Montgomerie 2004a; Herdman et al. 2004), as well as males in other species (Andersson 1994; Bonduriansky 2001; Clutton-Brock 2007; Edward and Chapman 2011), tend to prefer larger, more fecund females as mates. Preferentially mating with large and fecund females can potentially confer greater reproductive success to males that may offset the costs of mate choice (Parker 1983; Servedio and Lande 2006; Edward and Chapman 2012), subject to the constraints of sperm competition and cryptic female choice (Wedell et al. 2002).

The guppy is an important model species for the study of sexual selection (Houde 1997) and highly suitable for investigating male mating effort and mating preferences in the context of the evolution of male mate choice. This species is an internally fertilizing, ovoviviparous freshwater fish native to Trinidad that exhibits a resource-free promiscuous mating system and mutual mate choice. There is no parental care of young. Males achieve copulations and potential fertilizations either by soliciting a female using courtship sigmoid displays or circumventing female choice through sneak gonopodial thrusting (Houde 1997; Pilastro and Bisazza 1999); to be successful, both mating tactics require a male to socially associate (in close proximity) with females for varying amounts of time. The guppy system therefore meets several of the aforementioned conditions that favor the evolution of male mate choice. More specifically, in natural populations, individuals live in mixed-sex shoals wherein males encounter females simultaneously (Houde 1997; Jeswiet et al. 2011), adult females vary widely in quality (e.g., in body size, fecundity, and reproductive state/sexual receptivity; Reznick and Endler 1982; Houde 1997; Kelly et al. 1999; Herdman et al. 2004; Ojanguren and Magurran 2004), males experience intense mating and sperm competition (Kelly et al. 1999; Neff et al. 2008; Jeswiet et al. 2011), male mating effort is costly and highly plastic (e.g., Houde 1997; Ojanguren and Magurran 2004; Guevara-Fiore et al. 2010a; Head et al. 2010; Jeswiet and Godin 2011), females prefer males that exhibit high courtship rates (Houde 1997; Kodric-Brown and Nicoletto 2001), and the production of sperm ejaculates is rate limited (Pilastro and Bisazza 1999).

Our novel main findings, of positive covariation and high repeatability in mating effort and mating preference in male guppies, are consistent with the assumptions and predictions of recent models for the evolution of male mate choice (Servedio and Lande 2006; Rowell and Servedio 2009; South et al. 2012) and suggest that such directional mating effort and preference in male guppies select for large body size in females, have a genetic basis and are potentially responsive to selection and further evolution.

## Materials and Methods

### Subjects and general procedures

**Figure 1:**
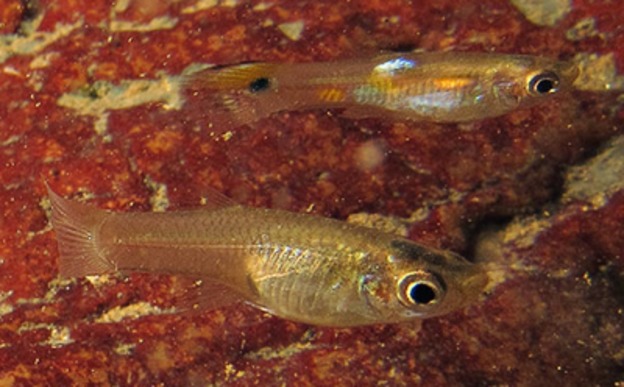
Photograph of free-ranging adult male (top) and female (bottom) guppies in the Naranjo tributary of the Upper Aripo River, Trinidad. Photo credit: P. Bentzen.

Our experimental guppies (Fig. 1) were wild adult fish collected haphazardly by hand seine from the Upper Aripo River (Naranjo tributary), a low-predation population (Magurran 2005), in Trinidad (10°41′70″N, 61°14′40″W) in April and May 2012. In this population, adult females vary widely in body size and fecundity, have broods that are multiply sired, and their fecundity is positively correlated with body length (Kelly et al. 1999; Herdman et al. 2004; Ojanguren and Magurran 2004; Neff et al. 2008).

Following field collections, the fish were transported to a laboratory at the University of the West Indies, St. Augustine and held in mixed-sex aquaria (at approximately 2 females: 1 male sex ratio; cf. Magurran 2005) filled with filtered aged tap water (22–25°C) and illuminated overhead with fluorescent lighting and diffused natural sunlight (entering the room via small windows near the ceiling). We fed them ad libitum twice daily with commercial flake food (Nutrafin™; Rolf C. Hagen, Inc., Montréal, Canada) and live brine shrimp nauplii (*Artemia franciscana*). As guppies can become familiar with each other after 12 days of association (Griffiths and Magurran 1997) and social familiarity can potentially affect male mate choice (Hughes et al. 1999; Kelley et al. 1999), the focal male and stimulus females used in any given mate choice trial were taken from different holding aquaria and were presumably unfamiliar with each other prior to testing.

The day before mate choice trials, males were isolated from females to allow them sufficient time to replenish their sperm reserves and to ensure that all test males were similarly sexually motivated at the onset of the behavioral trials. All females used in our study were at least 17 mm in standard length, and thus sexually mature (Houde 1997). Following Dosen and Montgomerie (2004b) and Jeswiet and Godin (2011), we importantly used gravid (pregnant) females, who are generally unreceptive to male courtship and copulation attempts (Houde 1997) and which we so confirmed here (see Results section), as stimulus fish to ensure that male mate choice would not be confounded by female sexual responses to male sexual activity and to minimize variation in male behavior caused by any differences in female reproductive state. Although male guppies generally prefer unmated over mated (gravid) females as mates when available (Guevara-Fiore et al. 2009, 2010b) and most adult female guppies in natural populations in Trinidad are pregnant at any given time (Houde 1997), males nonetheless sexually pursue, court, and attempt to mate with previously mated gravid females in both the wild and in the laboratory (Houde 1997; Guevara-Fiore et al. 2010b; Jeswiet et al. 2011). Female guppies can store viable sperm from multiple males for several months (Houde 1997) and exert apparent cryptic mate choice (Pilastro et al. 2002; Evans et al. 2003b). Despite risking sperm competition, males can successfully inseminate unreceptive gravid females through forced sneak copulations (Pilastro and Bisazza 1999; Evans et al. 2003a) and sire offspring (Kelly et al. 1999; Herdman et al. 2004; Neff et al. 2008).

### Mate choice experiment

To test for their mating effort and mating preference, we presented focal males with a simultaneous choice between two free-swimming stimulus females that were gravid, sexually nonreceptive and differing in body length, with which they could interact physically, in an open-field apparatus (see below) to reflect the natural mate-encounter conditions experienced by wild Trinidadian guppies (cf. Houde 1997; Guevara-Fiore et al. 2010b; Jeswiet et al. 2011). Thus, both the test male and the stimulus females had full access to each other and to all potential stimuli (visual, chemical, tactile) exchanged between them. Under this circumstance of unlimited sensory information, we assumed that males would to be able to accurately assess the differences in body size and reproductive state of the paired females (cf. Herdman et al. 2004; Hoysak and Godin 2007; Guevara-Fiore et al. 2010a,b).

Our open-field test apparatus consisted of a glass aquarium (40 × 20 × 25 cm; L × W × H), which had a substratum of natural river gravel, was filled with aerated aged water (15 cm depth, 23.5 ± 0.1°C), and illuminated overhead with fluorescent light tubes and diffuse natural sunlight. Three sides of the aquarium were covered externally with tan paper to provide a uniform visual background and reduce external disturbances. We observed the behavior of the fish through the open side of the aquarium from behind a blind between 08:00 and 17:00 h daily.

We repeatedly recorded the mating effort and mating preference of individual focal males for either of two stimulus females differing in body length on each of two consecutive days (i.e., paired trials 1 and 2), with the repeated trials 23.5–24.5 h (hereafter, 1 day) apart, as follows. Observations on the focal male in trial 2 were made blind of his behavior in trial 1. On the day of a trial, two gravid females were matched by eye as closely as possible for abdominal distension (and thus reproductive state; Houde 1997) and measured for body length without anesthesia (chosen to be different in length) using a metric scale. Each focal male was presented with different stimulus females in the paired repeated trials to avoid the possibility of male recognition of a particular female. The body lengths of the stimulus females used in trials 1 and 2 (*N* = 80, respectively) were very similar on average (Table S1). In any given trial, the paired stimulus females were chosen to similarly differ in body length on average by 4.8 mm (=23.7%) in trial 1 and 4.8 mm (=24.0%) in trial 2 (*t*-test, *t*_78_ = 0.206, *P* = 0.837; Table S1), to facilitate male mate choice based on female body size (cf. Dosen and Montgomerie 2004a; Herdman et al. 2004; Jeswiet and Godin 2011).

Prior to the onset of a trial, the test aquarium was temporarily divided in half with a clear, perforated plastic partition. A focal test male was placed on one side of this partition and two stimulus females differing in body length were placed on the other side. All fish were left undisturbed for 30 min to allow them to acclimatize to the aquarium and to view and smell each other across the partition. After this period, the partition was raised and the behavior of the fish was recorded live for 20 min. We changed the water in the test aquarium with fresh aged water after every completed trial. We similarly tested a total of 40 males individually.

Following Herdman et al. (2004) and Jeswiet and Godin (2011), we recorded the following male sexual behaviors (cf. Houde 1997) directed toward either stimulus female during each of the paired trials: (i) “approach,” an unambiguous directed movement of the male toward a female, (ii) “association time,” time during which a male actively follows within three body lengths a female with his head oriented toward her, (iii) “gonopodial nip,” mouth-nipping behavior by a male directed at a female's gonopore, (iv) “sigmoid display,” a courtship display directed at a female, which involves the male arching his body into an S-shape and quivering, and (v) “copulation attempt,” scored as a male approaching a female from the side or behind and rapidly thrusting his gonopodium forward toward her genital opening. At the end of each pair of repeated trials, the standard body length of the focal male was measured (Table S1), and the focal male and stimulus females were placed in separate holding aquaria and not reused.

We quantified the mating preference of each focal male in a given trial as the percentage of total association time spent near the larger female (= [association time with large female/sum of association times with small and large females] × 100), and male mating effort as the percentage of total sexual acts (excluding association time) directed at the larger female. Male association time with a particular female is a reliable predictor of mate choice in the guppy (Jeswiet and Godin 2011), as well as for other fishes (e.g., Walling et al. 2010). Because each of the sexual acts measured potentially contributes to male mating success in guppies (Houde 1997), they thus collectively represent a male's precopulatory mating effort (cf. Edward and Chapman 2011). Additionally, a male was categorically classified as “preferring” a particular female if he spent >50% of his total association time near her and concurrently directed >50% of his sexual acts (mating effort) toward her in a given trial (cf. Godin and Dugatkin 1995; Jeswiet et al. 2011, 2012).

### Statistical analysis

All statistical analyses were carried out in the R statistical software environment (R Development Core Team 2012) and all tests are two-tailed, unless specified otherwise. Not all data were normal in distribution. Therefore, to improve normality and homoscedasticity, percentage (proportion) data were arcsine transformed and data on counts were log_10_ transformed prior to analysis. The two main dependent behavioral variables of interest here (i.e., male mating effort, male mating preference) were normally distributed (Shapiro tests, all *P* > 0.492) and homoscedastic (Levene tests, all *P* > 0.298) following transformation.

We first tested the null hypothesis of no difference in the mating preference and mating effort of focal males for either of the paired stimulus females by comparing separately their mating preference (percent of association time with the larger female) and mating effort (percent of total sexual acts directed toward the larger female) scores, and the number of males categorized as preferring the larger female, against that expected by chance using the Wilcoxon signed-rank test and Binomial test, respectively. We then separately compared the frequencies of each of the recorded sexual acts directed by males toward the larger and smaller females using the Wilcoxon signed-rank test. We used linear mixed-effects models (LMMs) with the restricted maximum-likelihood (REML) method to test for any effects of trial number (trial 1 vs. trial 2), male body length and difference in the length of paired stimulus females on the mating preference and mating effort scores of focal males separately, controlling for male identity as a random variable in the models (Crawley 2007; Field et al. 2012).

Second, to characterize the relationship between male mating preference and mating effort, we correlated (i) the mating preference scores of individual males against their total mating effort (= total sexual acts) scores and (ii) mating preference scores against courtship effort scores (= percent of courtship displays exhibited toward the larger female) for each of the paired repeated trials separately using the Spearman rank correlation analysis. For this analysis, we necessarily excluded from the data set those individual behavioral trials (*N* = 9 out of 80 trials) in which the focal male did not court either stimulus females, but otherwise exhibited all the other sexual acts toward them.

Finally, we separately calculated the repeatability of male mating preference and mating effort scores between paired trials 1 and 2 using LMMs with the REML method in the “rptR” package (“rpt.remlLMM” function; Nakagawa and Schielzeth 2010) developed in R software (R Development Core Team 2012), given that the data were Gaussian. In the models, fish identity was assigned as a grouping random factor (Nakagawa and Schielzeth 2010). For each estimated repeatability (R) coefficient, we provided the associated calculated standard error (SE) and 95% confidence interval (CI). To ascertain whether the mating preference or mating effort score of a focal male in trial 1 would predict his preference or mating effort score, respectively, 1 day later in trial 2, we regressed separately (using simple linear regression analysis) each of these two behavioral measures obtained for trial 1 against that for paired trial 2.

## Results

All focal males used were sexually active and directed mating effort toward both paired stimulus females. As expected, the stimulus females used did not exhibit any obvious sexual behavior toward the focal males in the current study (because they were gravid and thus unreceptive to male sexual solicitations; cf. Houde 1997).

On average, males exhibited significantly greater mating effort toward, and spent more time associating with, the larger female than expected by chance in both paired trials (Wilcoxon signed-rank test, all *P* < 0.0001, Fig. S1). Therefore, significantly more males than expected by chance (33 out of 40 in Trial 1, and 32 of 40 in Trial 2) categorically preferred the larger of the two stimulus females (one-tailed Binomial test, both *P* < 0.0001). Considering the constituent components of mating effort, males exhibited significantly more approaches, sigmoid courtship displays, gonopodial nips, and copulation attempts toward the larger of the paired stimulus females (Wilcoxon signed-rank test, all *P* < 0.0001, Fig. S2).

**Figure 2:**
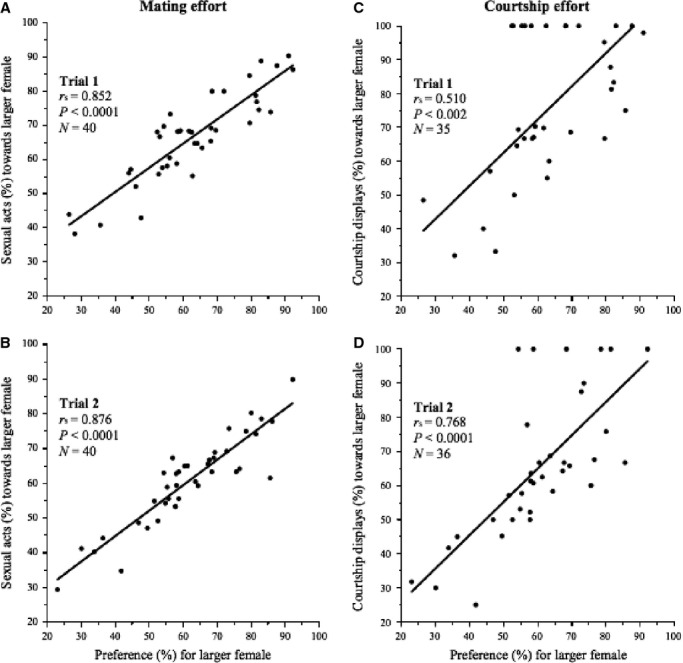
Relationships between the mating preference of focal males, based on percent association time with the larger female, and their mating effort (panels A and B) and courtship effort (panels C and D) for paired repeated trials 1 and 2. The best-fit lines were obtained using simple linear regression analysis. The correlation coefficient (*r*_*s*_) and *P* values shown were obtained using the Spearman rank correlation analysis.

Although males preferred larger females on average, the scores for two measured proxy components of their mate choice (mating preference and mating effort) varied widely among individuals and were significantly positively correlated with each other in both repeated trials (Fig. 2A, B). Similarly, a significant positive relationship between mating preference and courtship effort (a component of mating effort) was also observed in both repeated trials (Fig. 2C, D). Neither mating preference nor mating effort scores of individual males were influenced by their body length (LMMs, *t*_38_ = 1.28, *P* = 0.209; *t*_38_ = 1.20, *P* = 0.237, respectively) or the difference in the body length of the paired stimulus females (LMMs, *t*_38_ = 1.05, *P* = 0.301; *t*_38_ = 0.52, *P* = 0.603, respectively).

**Figure 3:**
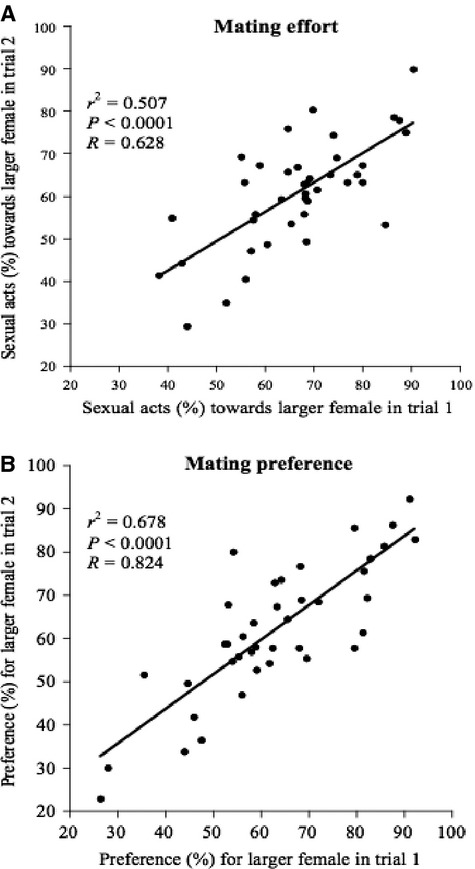
Relationships between the mating effort scores of focal males, based on the percent of sexual acts directed toward the larger female (panel A), and their mating preference scores, based on percent association time with the larger female (panel B), during trial 1 and their scores 1 day later in repeated trial 2. The best-fit lines, and associated *r*^2^ and *P* values, were obtained using simple linear regression analysis. Repeatability (*R*) estimates were obtained using the linear mixed-effects model with the restricted maximum-likelihood (REML) method described in Nakagawa and Schielzeth (2010).

The mating effort and mating preference of individual males were also both highly repeatable between the paired trials, as measured by sexual acts directed toward the larger female (repeatability estimate*, R* = 0.628 ± 0.099, 95% CI = 0.403–0.786, *P* < 0.0001; Fig. 3A) and by association time with the larger female (*R* = 0.824 ± 0.053, 95% CI = 0.703–0.905, *P* < 0.0001; Fig. 3B), respectively. That is, there was significant variation in both mating effort and mating preference between subjects, and individual subjects were consistent in these sexual behaviors over time.

## Discussion

Although male mate choice does occur in nature, it is not as well understood as female mate choice (Andersson 1994; Amundsen 2000; Bonduriansky 2001; Clutton-Brock 2007; Edward and Chapman 2011). Because male mate choice is more likely to evolve when individual variation in mating effort and mating preference exists, and when male courtship effort (a component of mating effort) and mating preference positively covary (Servedio and Lande 2006; Rowell and Servedio 2009; Edward and Chapman 2011; South et al. 2012), understanding such phenotypic variation is critical for understanding sexual selection and the evolution of mate choice (Jennions and Petrie 1997; Widemo and Sæther 1999; Edward and Chapman 2011). In this context, the results of our current study support the assumptions and predictions of recent evolutionary models of male mate choice (Servedio and Lande 2006; Rowell and Servedio 2009; South et al. 2012) and thus advance our understanding of the importance of phenotypic variation (in mating effort and preference) within natural populations in the evolution of male mate choice.

Here, we showed that male Trinidadian guppies on average directed significantly greater mating effort and exhibited a preference for (as measured by association time with) the larger of two females, independent of female identity, when presented concurrently in an open-field arena. The observed preferences cannot be explained by any differential sexual behavior of the (gravid) stimulus females toward focal males, as they were generally sexually unreceptive to males (cf. Houde 1997), nor by variation in male body length or body length difference between paired stimulus females. These results generally corroborate those of Abrahams (1993), Dosen and Montgomerie (2004a), Herdman et al. (2004), Head et al. (2010) and Jeswiet et al. (2012), on guppies of different provenances than ours, and thus collectively demonstrate that male guppies possess a generalized mating preference for large females. Male allocation of greater mating effort toward, and preference for, larger and more fecund females as mates is fairly widespread taxonomically (Andersson 1994; Bonduriansky 2001; Clutton-Brock 2007; Edward and Chapman 2011). Preferring to mate with large females would appear to be adaptive, as large female guppies are more fecund (Reznick and Endler 1982; Kelly et al. 1999; Herdman et al. 2004; Ojanguren and Magurran 2004) and thus of potentially greater reproductive value to males than smaller ones (Parker 1983; Andersson 1994; Edward and Chapman 2012), all else being equal.

However, if all males have an equal amount of resources that they can allocate to courtship (or mating effort in general) and similarly bias the distribution of this effort toward preferred females in polygynous or promiscuous systems, then male choice expressed as increased courtship toward preferred females can lead to increased male mating competition for the most attractive females (and potentially increased sperm competition, Wedell et al. 2002) in the population and consequently to a loss of a male preference allele (Servedio and Lande 2006). This cost of increased competition for preferred females, which constrains the evolution of male mate choice, can be offset or mitigated if (i) preferred females have sufficiently higher fecundity or mating success (Parker 1983; Servedio and Lande 2006; Edward and Chapman 2012), (ii) males can avoid or minimize sperm competition by adjusting their mating effort and preference accordingly (Wedell et al. 2002; Rowell and Servedio 2009), and(or) (iii) courtship is costly and males differ in how they distribute their courtship effort among females and females prefer males that exhibit high courtship effort (Servedio and Lande 2006; Rowell and Servedio 2009; South et al. 2012).

The latter strategic conditions favoring the evolution and maintenance of male mating preferences in populations exist in the guppy mating system. More specifically, male guppies exhibit on average a mating preference for larger females (current study; Abrahams 1993 Herdman et al. 2004; Head et al. 2010; Ojanguren and Magurran 2004; Jeswiet et al. 2011), female fecundity is strongly positively correlated with their body size (Reznick and Endler 1982; Kelly et al. 1999; Herdman et al. 2004; Ojanguren and Magurran 2004), and larger multiply-mated females produce larger (and presumably more viable) offspring than smaller females (Ojanguren et al. 2005). Male courtship behavior is costly (Godin 1995; Head et al. 2010) and an honest, condition-dependent indicator of male quality (Nicoletto 1993; Houde 1997; Matthews et al. 1997; Kolluru et al. 2009), and females prefer males exhibiting with high courtship effort, at least in some populations (Houde 1997; Kodric-Brown and Nicoletto 2001). Moreover, male guppies are sensitive to the local risk of sperm competition and adaptively reduce their mating preferences for larger, more fecund females and redirect their mating effort toward smaller, less fecund females in response to a perceived increase in the risk of sperm competition associated with larger, more attractive females (Dosen and Montgomerie 2004b; Jeswiet et al. 2011, 2012), thereby potentially contributing to the maintenance of variation in male mating preferences in the population. Finally, as we demonstrated in the current study, males vary widely and consistently in their mating effort (including courtship) and mating preference for females based on body size, and individual male mating effort (including courtship effort) and mating preferences are strongly, positively correlated.

Phenotypic and genetic variation in traits is required for selection (Lynch and Walsh 1998), and variation owing to individual plasticity in mating behavior may be adaptive under a range of conditions (Qvarnström 2001; Bretman et al. 2011). Although we and other investigators have previously observed variation among male guppies in their mating preferences based on female body size (Abrahams 1993; Dosen and Montgomerie 2004a,b; Jeswiet and Godin 2011; Jeswiet et al. 2011, 2012), our current study is the first to comprehensively characterize and analyze individual variation in both male mating effort and mating preference and to report on their repeatabilities for any species (cf. Bell et al. 2009) to our knowledge. We showed that mating effort and mating preference in wild-caught male guppies positively covary and are significantly more variable among than within individuals, and that individual males are thus consistent, but not unanimous, in their mate choice (cf. Widemo and Sæther 1999), at least in our study population. The nature of such phenotypic variation in mating effort and preference would maintain male mate choice, once evolved, within the population (cf. Servedio and Lande 2006; Edward and Chapman 2011). To the extent that repeatability of a trait places an upper limit on its heritability (Lynch and Walsh 1998), the observed high repeatabilities for mating effort and mating preference, based on female body size, obtained for Upper Aripo River male guppies here is consistent with the presence of additive genetic variation for both these traits in this population and thus with the opportunity for selection and further evolution of large female body size through male mate choice. There is additionally some limited evidence for a genetic basis to male courtship effort in the guppy (Nicoletto 1995; Mariette et al. 2006). However, the evolutionary exaggeration of female body size in the guppy, under directional male mate choice, is constrained by resource limitation, life-history trade-offs and costs associated with large body size in females (Magurran 2005) and by sperm competition (Kelly et al. 1999; Dosen and Montgomerie 2004b; Neff et al. 2008; Jeswiet et al. 2011, 2012) and polymorphism in the allocation of mating effort and mating preference by males among females in the population (current study).

Given the relative paucity of studies on variation in male mate choice (Bell et al. 2009; Edward and Chapman 2011), our current study thus represents an important contribution to further characterizing and understanding variation in male mate choice and its evolution within natural populations. Enduring challenges include understanding the genetic and environmental bases of individual variation in male mating effort and mating preference, the relationship between individual variation in mate choice and variance in lifetime reproductive success among males, and the interactions between male and female mate choice on sexual selection in species with mutual mate choice (Edward and Chapman 2011). Because it exhibits mutual mate choice, male reproductive skew (Neff et al. 2008), and within-population variation in repeatable mating preferences in both males (current study) and females (Godin and Dugatkin 1995), the Trinidadian guppy offers a particularly suitable model species for pursuing these lines of investigation in the future.

## Supporting Information

**Figure S1.** Mating effort and mating preference of focal males for the larger female in each of the repeated trials. Mating effort is depicted as the mean ± SE percent of total sexual acts directed by focal males toward the larger female, and mating preference as the mean ± SE percent time focal males spent associating with the larger female. The horizontal line denotes random choice between the paired stimulus females. The asterisk above individual histogram bars indicates a significant (*P* < 0.0001; Wilcoxon signed-rank test) difference between the observed mean score and that expected from chance (i.e., 50%) for that particular trial. The *P*-value above each pair of histogram bars (paired trials 1 and 2) was obtained using linear mixed-effects models testing for any effect of trial number on the behavioral scores of males in each experiment.

**Figure S2.** Male mating effort expressed as the mean ± SE frequency (number/40 min) of each of the different component sexual acts, and their total sum, directed by focal males toward each of the paired stimulus females over the course of the repeated paired trials. AP, approach; SD, courtship sigmoid display; GN, gonopodial nip; CA, copulation attempt. ****P* < 0.0001, obtained using the Wilcoxon signed-rank test.

**Table S1.** Mean ± SE (range) standard body lengths of the focal males and paired stimulus females used on repeated consecutive days (paired trials 1 and 2), and the mean ± SE absolute and relative differences in the standard body length of the paired stimulus females used in individual behavioral trials. Individual focal males were tested repeatedly in paired trials 1 and 2.
