# Supplementary figures and images for: Covariation and repeatability of male mating effort and mating preferences in a promiscuous fish
Source: Ecol Evol. 2013 May 31;3(7):2020–9. doi: 10.1002/ece3.607 (PMC3728943; doi:10.1002/ece3.607)

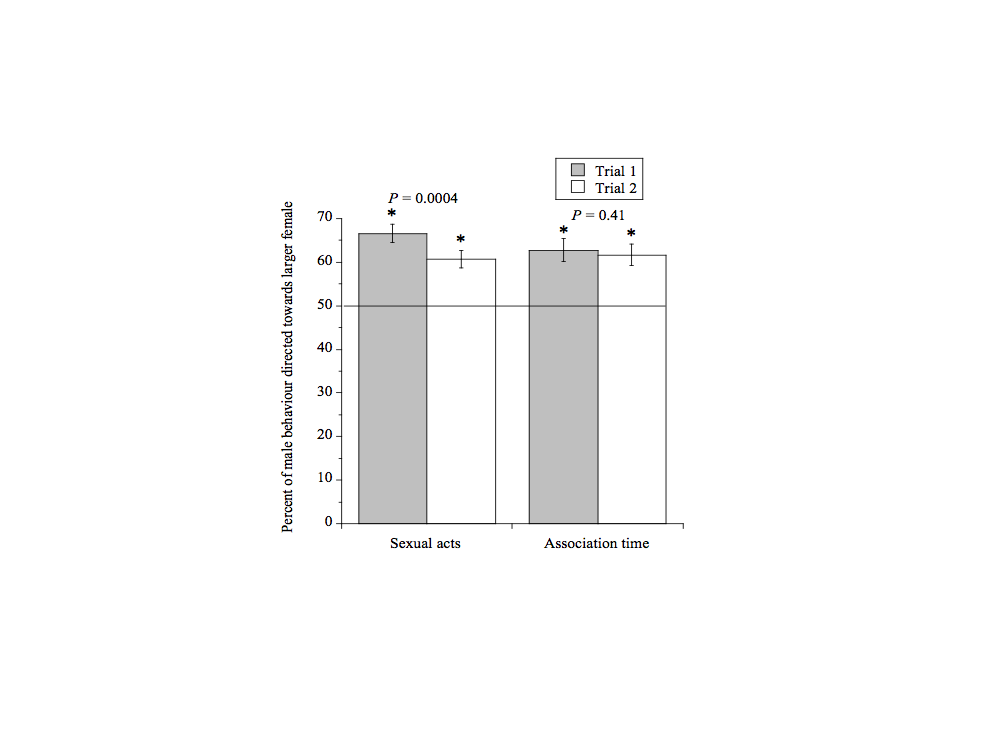

Supplement: Supplementary file 1 [file ece30003-2020-SD1.tif]

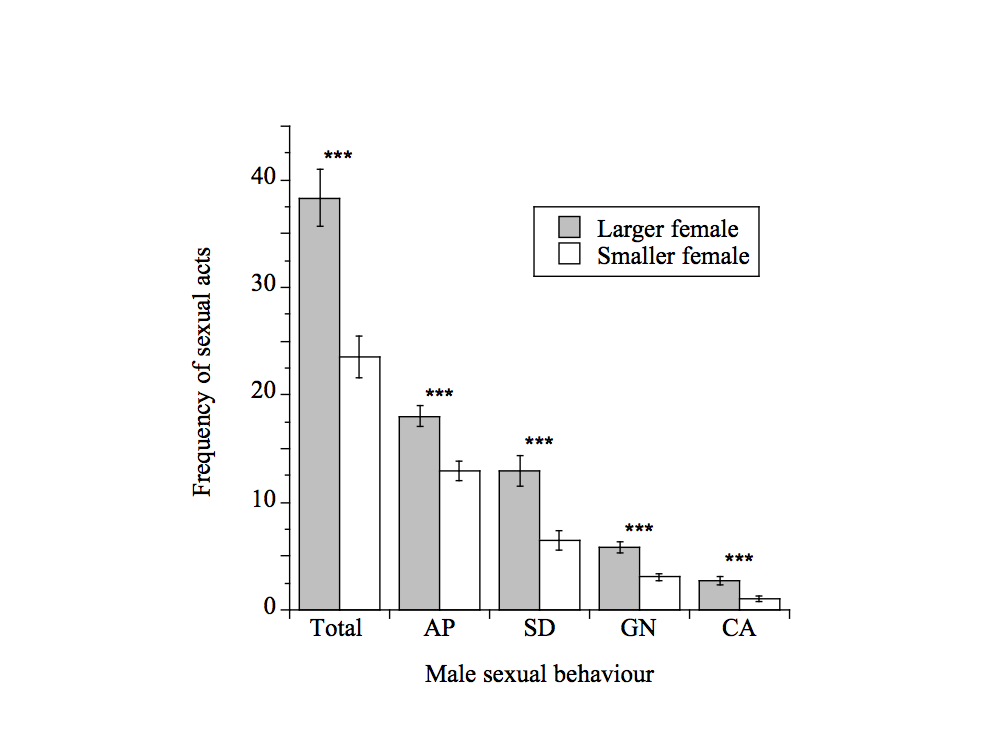

Supplement: Supplementary file 2 [file ece30003-2020-SD2.tif]

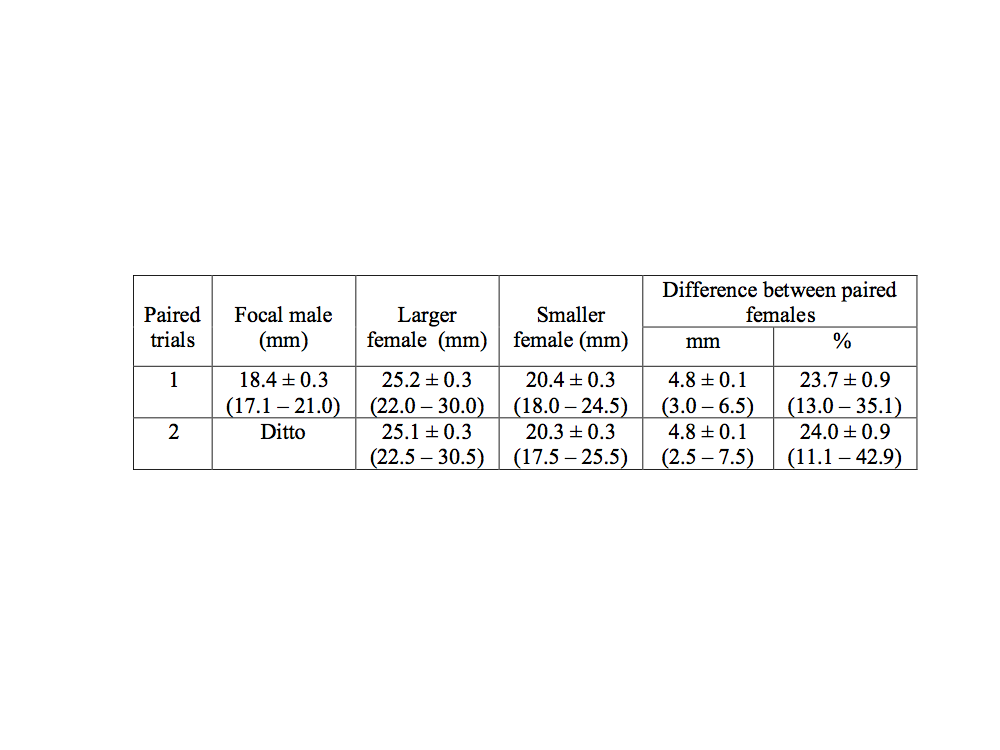

Supplement: Supplementary file 3 [file ece30003-2020-SD3.tif]
